# 941. Improving Burn Care: Reducing Missed ED Consults Through Real-time Monitoring and Multidisciplinary Collaboration

**DOI:** 10.1093/jbcr/irag033.508

**Published:** 2026-04-06

**Authors:** Sydney Mullins, Erin Rhinehart, Kathleen S Romanowski, Tina L Palmieri, Soman Sen, Jason Heard, David Barnes, Alessandra Renteria-Turley

**Affiliations:** Firefighters Burn Institute Regional Burn Center - University of California Davis, Rancho Cordova, CA; University of California Davis Health, West Sacramento, CA; Shriners Children's - Northern California, Sacramento, CA; University of California Davis Health, Shriners Children’s - Northern California, Sacramento, CA; Shriners Children's - Northern California, Sacramento, CA; Shriners Children's - Northern California, Sacramento, CA; University of California Davis Health, Sacramento, CA; University of California Davis Health, Sacramento, CA

## Abstract

**Introduction:**

Timely evaluation by burn-trained specialists is critical to optimize outcomes for burn-injured patients. However, our institution identified a concerning pattern: patients presenting to the Emergency Department (ED) with burn injuries were often not being evaluated by the Burn Surgery team. As a result, these individuals were discharged without appropriate follow-up plans, placing them at risk for complications, delayed healing, and poor functional or cosmetic outcomes.

**Methods:**

To address this care gap, a daily report was implemented to identify all ED patients presenting with burn injuries. Any case lacking notification to the burn team is escalated in real time to the Burn Center Director, Nurse Manager, and Burn Surgery team for evaluation and prioritization for outpatient follow-up. Missed consult opportunities are also communicated directly to our ED liaison team for immediate process improvement. Additionally, all identified cases are compiled and reviewed monthly, with comprehensive discussion at the Burn Center’s Quarterly QA/QI Meeting by the multidisciplinary team. Education was also provided to ED providers regarding the importance of consulting the Burn Surgery team when burn-injured patients present to the ED.

**Results:**

As a result of these combined interventions, the percentage of missed ED consults decreased significantly, from 28.6% of burn-injured patients not being seen by the Burn Surgery team in calendar year (CY) 2020 to 9.9% in CY 2025. This demonstrates a measurable improvement in care coordination and specialty capture for burn patients.

**Conclusions:**

This proactive, data-driven approach has enhanced the identification and management of burn-injured patients in the ED. It has also fostered accountability and communication between emergency and specialty teams, creating a more reliable pathway to ensure timely burn care.

**Applicability of Research to Practice:**

Our experience highlights the critical role of real-time monitoring, provider education, and interdisciplinary escalation pathways in improving patient outcomes. This model is highly replicable and could be adapted by other institutions seeking to close gaps in specialty consults and improve continuity of care for vulnerable patient populations.

**Funding for the study:**

N/A.

